# Effects of the increased protein level in small intestine on the colonic microbiota, inflammation and barrier function in growing pigs

**DOI:** 10.1186/s12866-022-02498-x

**Published:** 2022-07-06

**Authors:** Zhongxin Li, Liren Ding, Weiyun Zhu, Suqin Hang

**Affiliations:** 1 National Center for International Research On Animal Gut Nutrition, Jiangsu Key Laboratory of Gastrointestinal Nutrition and Animal Health, Laboratory of Gastrointestinal Microbiology, Nanjing Agricultural University, No.1 Weigang, Region Xuanwu, Nanjing, 210095 Jiangsu China; 2 National Experimental Teaching Center for Animal Science, College of Animal Science and Technology, Nanjing Agricultural University, Nanjing, 210095 China

**Keywords:** Soy protein hydrolysate, Growing pig, Colonic microbiota, Metabolic profile, Inflammatory cytokines, Tight junction proteins

## Abstract

**Background:**

An increased level of the dietary protein alters the colonic microbial community and metabolic profile of pigs, but it remains unclear whether this leads to colonic inflammation and impairs barrier function in growing pigs.

**Results:**

Sixteen pigs (35.2 ± 0.3 kg) were infused with sterile saline (control) or soy protein hydrolysate (SPH) (70 g/day) through a duodenal fistula twice daily during a 15-day experimental period. The SPH treatment did not affect their average daily feed intake and daily weight gain (*P* > 0.05), but reduced colon index and length (*P* < 0.05). Illumina MiSeq sequencing revealed that species richness was increased following SPH intervention (*P* < 0.05). Furthermore, SPH reduced the abundance of butyrate- and propionate-producing bacteria—such as *Lachnospiraceae NK4A136 group*, *Lachnospiraceae_uncultured*, *Coprococcus 3*, *Lachnospiraceae UCG-002*, and *Anaerovibrio*—and increased the abundance of potentially pathogenic bacteria and protein-fermenting bacteria, such as *Escherichia-Shigella*, *Dialister*, *Veillonella*, *Prevotella*, *Candidatus Saccharimonas*, *Erysipelotrichaceae UCG-006*, *Prevotellaceae_uncultured*, and *Prevotellaceae UCG-003* (*P* < 0.05). In addition, a lower content of total short-chain fatty acids, propionate, and butyrate and a higher concentration of cadaverine, putrescine, total biogenic amines, ammonia, and isovalerate were observed following SPH infusion (*P* < 0.05). Further analysis revealed that SPH increased the concentration of tumour necrosis factor-α, interleukin (IL)-1β, IL-6, and IL-8 in the colonic mucosa (*P* < 0.05). Interestingly, SPH intervention increased the expression of occludin, zonula occludens (ZO)-1, and claudin-1 in colonic mucosa (*P* < 0.05). Correlation analysis showed that different genera were significantly related to the production of metabolites and the concentrations of pro-inflammatory cytokines.

**Conclusion:**

An increased soy protein level in the small intestine altered the colonic microbial composition and metabolic profile, which resulted in the secretion of colonic proinflammatory cytokines and the increased expression of tight junction proteins.

**Supplementary Information:**

The online version contains supplementary material available at 10.1186/s12866-022-02498-x.

## Introduction

Gut health has substantial far-reaching impact on the overall good health of pigs and is affected by numerous factors, such as nutrition, environment, and genetics. Among these, nutritional factors (protein, fat, and carbohydrate levels) play a major role in a pig’s gut health [1]. Protein is an indispensable nutrient for the growth of pigs and increased dietary protein levels can improve a pig’s growth performance [2]. However, protein metabolism is generally considered detrimental to the host colon, because the distal colon—primarily where bowel disease begins—is also the main site of protein fermentation [3].

Excess dietary protein along with peptides and free amino acids that escape absorption in the small intestine enter the large intestine and are mainly fermented by abundant microbes colonized in the colon [4]. In a rat experiment, high-protein diets (HPD) promoted protein fermentation in the rat’s colon and the production of protein metabolites was found to be significantly positively correlated with colon inflammation and barrier function damage [5]. Another study revealed that an increased protein level in the colon increased the risk of diseases (like colon inflammation) in healthy people and potential pathogens in human faecal samples and reduced the abundance of total bacteria and beneficial bacteria (like the butyrate-producing *Roseburia/Eubacterium rectale* group) [6]. Therefore, colonic microbial disorders and changes in metabolic patterns may be the main reasons for the occurrence of colon diseases due to the increased protein levels. Previous studies reported that increased intestinal protein levels altered the composition of colon microbiota in piglets [7, 8]. The colonic microbiome of pigs can ferment protein in vitro to produce various protein metabolites, such as biogenic amines and branched chain amino acids [9]. However, it remains unclear whether the increased level of protein in the intestine leads to colonic inflammation and impairs barrier function by affecting the microbiota and its metabolic patterns in growing pigs. The intestinal structure and microbial composition differed among the species (humans, rodents, and pigs) and more investigations are required in pigs.

Soy protein is the main dietary protein for pigs and is considered a high-quality protein derived from soybeans. In this study, soy protein was selected and hydrolysed into soy protein hydrolysate (SPH), which was then injected into a pig’s duodenum through a fistula. This model was used in the early stage of our laboratory to explore the effect of SPH on the secretion of intestinal satiety hormones [10] and it was further used in this study for our specific objectives.

We hypothesized that an increased level of soy protein in small intestine will alter the colonic microbiota and its metabolic profile, thereby causing colonic inflammation and impairing barrier function in growing pigs. Thus, we utilized the fistula model to explore the effects of SPH infusion on the microbial community composition and metabolites as well as the expression of tight junction proteins and inflammatory cytokines in the colon of growing pigs.

## Results

### Growth performance and colon index

Table 1 demonstrates that the average daily feed intake, average daily gain, and ratio of feed to gain were not markedly different between the saline and SPH treatment pigs (*P* > 0.05). The protein levels of the saine group (18.0% protein) and the SPH group (21.4% protein) were calculated based on the dietary composition (Table 2) and average daily feed intake. Colon index and colon length were lower in the SPH group relative to the saline group (*P* < 0.05).

**Table 1:** Growth performance and colon index in the saline and SPH groups. Values are mean ± SEM (*n* = 8).* P* < 0.05 implies statistically significant. Colon index = (colon weight/body weight) × 100%

Items	Saline	SPH	*P-*value
Average daily feed intake (kg/d)	1.73 ± 0.07	1.61 ± 0.07	0.236
Average daily gain (kg)	0.87 ± 0.04	0.75 ± 0.06	0.095
Feed conversion ratio	2.06 ± 0.10	2.13 ± 0.18	0.737
Colon index (%)	4.57 ± 0.08	3.77 ± 0.29	0.020
Colon length (m)	3.71 ± 0.12	3.27 ± 0.11	0.016

Values are mean ± SEM (*n* = 8).* P* < 0.05 implies statistically significant. Colon index = (colon weight/body weight) × 100%

**Table 2:** Dietary composition and nutritional component (air dry basis)

Items	Composition, %
Ingredients	
Corn	59.81
Soya bean meal	25.80
Wheat flour	5.00
Rice bran meal	3.00
Corn germ meal	3.00
L-Lysine, 98.5%	0.11
DL-Methionine, 99.0%	0.04
L-Threonine, 99.0%	0.04
L-Tryptophan, 99.0%	0.01
Calcium hydrogen phosphate	0.40
Limestone	1.00
NaCl	0.50
Acidifying agent	0.40
Zinc oxide	0.20
Choline chloride	0.10
Mould inhibitor	0.05
Phytase	0.02
Compound enzyme	0.02
Mineral premix^a^	0.50
Nutrition level, %	
NE, MJ/Kg	10.19
Crude protein	18.00
Crude fat	2.63
Crude fiber	3.79
Crude ash	5.03
Lysine	1.10
Methionine	0.34
Tryptophan	0.21
Threonine	0.75
Valine	0.87

^a^Premix supplied the following per kg complete diet: vitamin A, 3,800 IU; vitamin D_3_, 800 IU; vitamin E, 9 mg; vitamin B_1_, 1 mg; vitamin K_3_, 1 mg; vitamin B_2_, 2 mg; vitamin B_6_, 1.2 mg; vitamin B_12_, 10 µg; nicotinic acid, 10 mg; biotin, 50 µg; folic acid, 0.4 mg; iron (as FeSO_4_ · H_2_O), 80 mg; zinc, 80 mg; iodine (as KI), 0.14 mg; Se (as Na_2_SeO_3_), 0.25 mg; copper as (CuSO_4_ · 5H_2_O), 5 mg; and Mn (as MnSO_4_ · H_2_O), 3 mg

### Effect of SPH on colonic microbiota

**Fig. 1:**
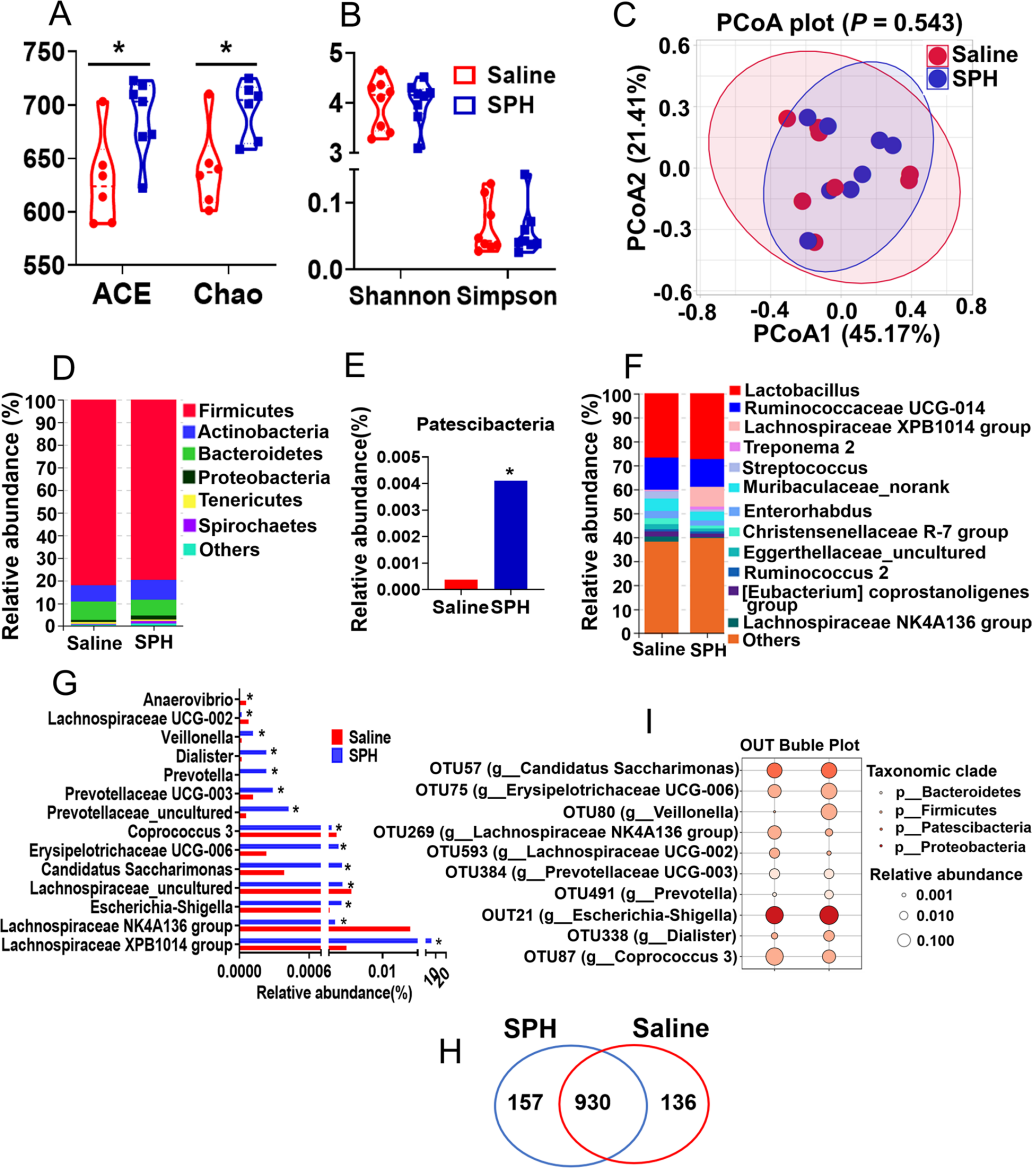
Changes in the microbial composition of colonic digesta with SPH treatment (*n* = 8). **A** The abundance-based coverage estimator (ACE), Chao index, and (**B**) the Simpson and Shannon index of colonic microbiota. **C** Principal coordinates analysis plots (PCoA) of colonic microbiota. **D** Relative abundance of the microbial phylum and (**E**) different phyla (Mann–Whitney U-test; median values). **F** Relative abundance of the microbial genus and (**G**) different genera (Mann–Whitney U-test; median values). **H** Analysis of the Venn diagram reveals the total number of OTUs both shared and unique. **I** Significantly different OTUs bubble chart. * *P* < 0.05

Compared with the saline group, the abundance-based coverage estimator (ACE) and Chao index representing species richness were increased in the SPH group (*P* < 0.05, Fig. 1A). The Shannon and Simpson indexes related to species diversity did not differ between the SPH and saline groups (*P* > 0.05, Fig. 1B). PCoA showed no difference in microbiota composition between the two groups (*P* > 0.05, Fig. 1C).

At the phylum level, *Firmicutes* and *Actinobacteria* were the two predominant phyla, contributing 74.36% and 6.49% of colonic microbiota in the saline treated pigs and 75.08% and 8.25% in the SPH treated pigs, respectively. *Bacteroidetes* and *Proteobacteria* constituted the next two most dominant phyla at 7.24% and 0.95% in saline treated pigs and 6.37% and 1.76% in SPH treated pigs, respectively (Fig. 1D). However, SPH treatment increased the abundance of phyla *Patescibacteria* (*P* < 0.05, Fig. 1E). At the genus level, *Lactobacillus*, *Ruminococcaceae UCG-014*, *Lachnospiraceae XPB1014 group*, *Treponema 2*, and *Streptococcus* were the abundant genera (Fig. 1F). It also revealed that the abundance of genra *Lachnospiraceae XPB1014 group*, *Veillonella*, *Prevotella*, *Candidatus Saccharimonas*, *Erysipelotrichaceae UCG-006*, *Prevotellaceae_uncultured*, *Escherichia-Shigella*, *Dialister*, and *Prevotellaceae UCG-003* was higher in the SPH group, whereas the abundance of the *Lachnospiraceae NK4A136 group*, *Lachnospiraceae_uncultured*, *Coprococcus 3*, *Lachnospiraceae UCG-002*, and *Anaerovibrio* was lower in the SPH group relative to the saline group (*P* < 0.05, Fig. 1G).

A total of 1223 OTUs were detected. Nine hundred and thirty OTUs of them were shared between the two groups, and 136 OTUs were unique in the saline treated pigs and 157 OTUs were unique in the SPH treated pigs (Fig. 1H). Among the different OTUs, 10 OTUs underwent transformation corresponding to the genera affected by the SPH. Specifically, in comparison with the saline group (*P* < 0.05, Fig. 1I), the SPH group showed a lower abundance of OTU87 (g__*Coprococcus 3*), OTU269 (g__*Lachnospiraceae NK4A136 group*), and OTU593 (g__*Lachnospiraceae UCG-002*) and greater abundances of OTU57 (g__*Candidatus Saccharimonas*), OTU75 (g__*Erysipelotrichaceae UCG-006*), OTU80 (g__*Veillonella*), OTU384 (g__*Prevotellaceae UCG-003*), OTU491 (*g__Prevotella*), OUT21 (*g__Escherichia-Shigella*), and OTU338 (*g__Dialister*) (*P* < 0.05).

### Colonic microbial metabolites

**Fig. 2:**
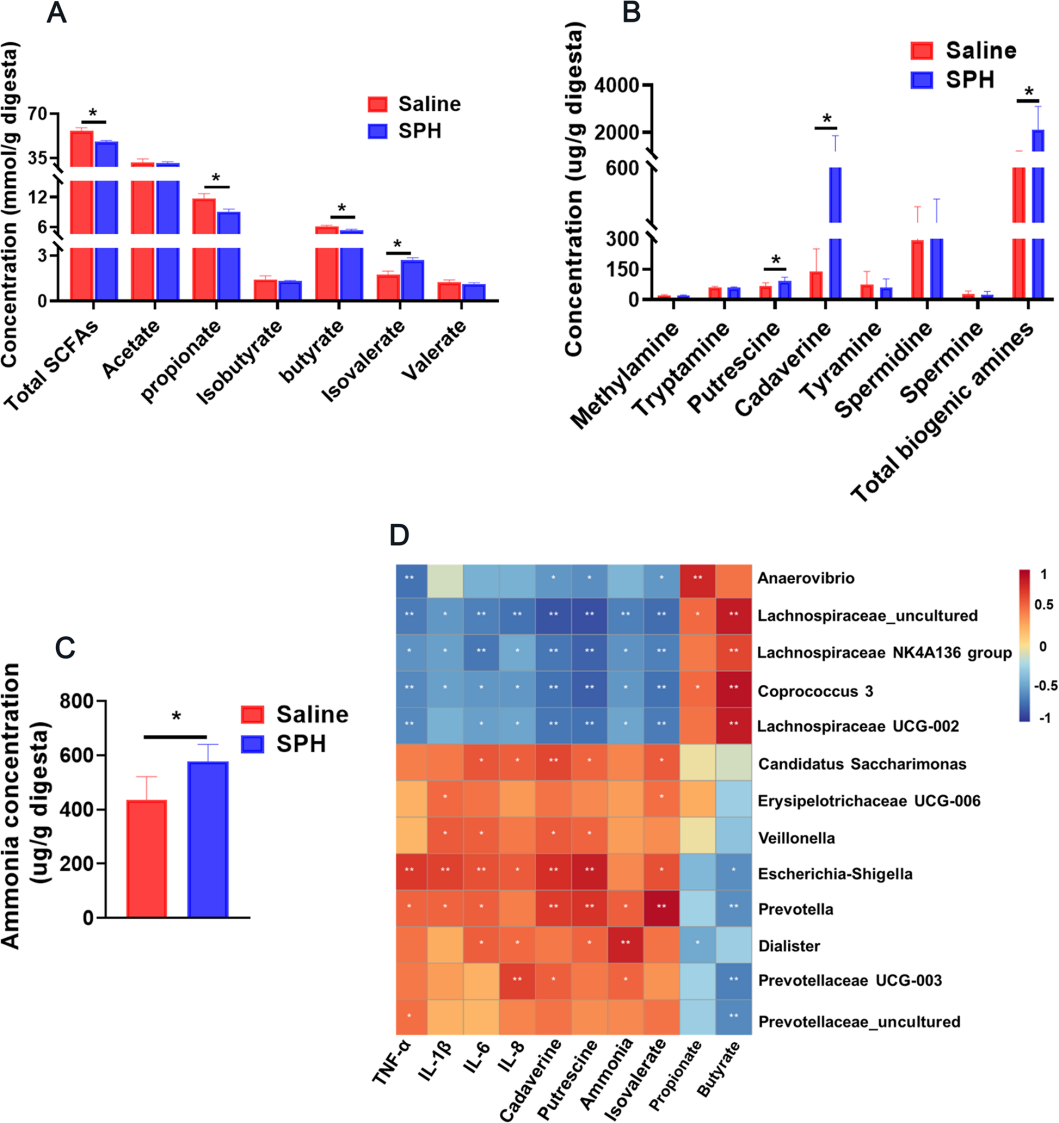
Colonic microbial metabolites and correlation analysis (*n* = 8). **A** Concentrations of SCFAs, (**B**) biogenic amines, and (**C**) ammonia in the colonic digesta of saline and SPH pigs. Values are mean ± SEM. **D** Spearman’s correlation analysis of different genera with metabolites and inflammatory cytokines. * *P* < 0.05 and ** *P* < 0.01

The concentration of SCFAs, biogenic amines, and ammonia was determined as an indicator of microbial fermentation. Compared with the saline group, a higher content of isovalerate and lower content of propionate, butyrate, and total SCFAs were observed in the SPH group (*P* < 0.05, Fig. 2A). Meanwhile, it observed that SPH infusion increased the content of cadaverine, putrescine, and total biogenic amines (*P* < 0.05, Fig. 2B). The ammonia concentration of the SPH group was higher than that of the saline group as well (*P* < 0.05, Fig. 2C).

Spearman’s correlation analysis was performed on different genera and metabolites (Fig. 2D). The five genera, *Veillonella*, *Prevotella*, *Candidatus Saccharimonas*, *Escherichia-Shigella*, and *Dialister*, markedly increased in abundance of the SPH group were positively correlated with the colonic concentration of ammonia, isovalerate, cadaverine, and putrescine (*P* < 0.05); the five genera, *Lachnospiraceae NK4A136 group*, *Lachnospiraceae_uncultured*, *Coprococcus 3*, and *Lachnospiraceae UCG-002*, decreased in abundance of the SPH group were positively correlated with the content of butyrate (*P* < 0.05). Genera *Anaerovibrio* was positively correlated with the content of propionate (*P* < 0.05).

### Concentration of inflammatory cytokines and expression of tight junction proteins

**Fig. 3:**
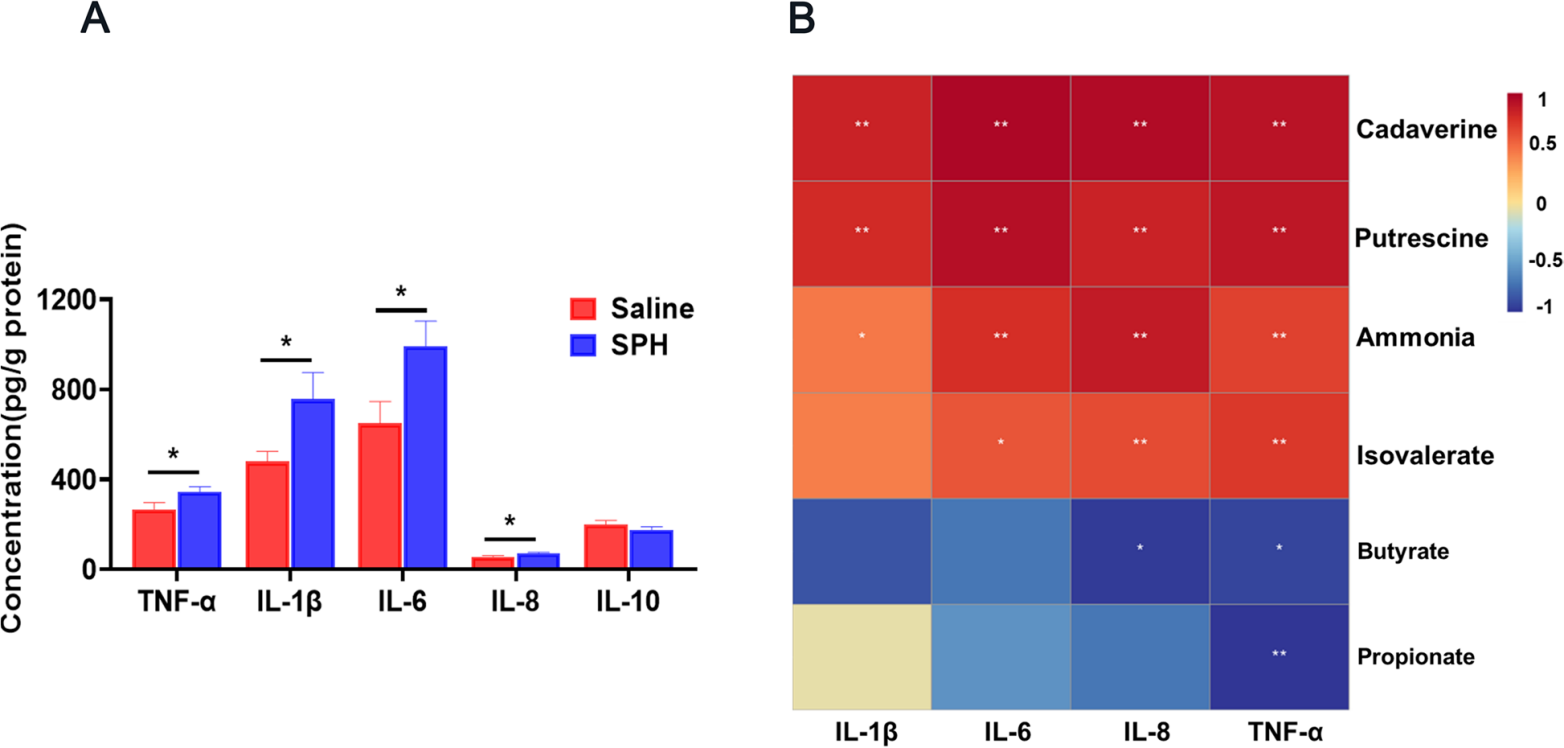
Inflammatory cytokines and correlation analysis (*n* = 8). **A** Concentrations of inflammatory cytokines in colonic mucosa in saline and SPH groups. Values are mean ± SEM. **B** Spearman’s correlation analysis between the concentration of metabolites and the concentration of inflammatory cytokines. * *P* < 0.05 and ** *P* < 0.01

**Fig. 4:**
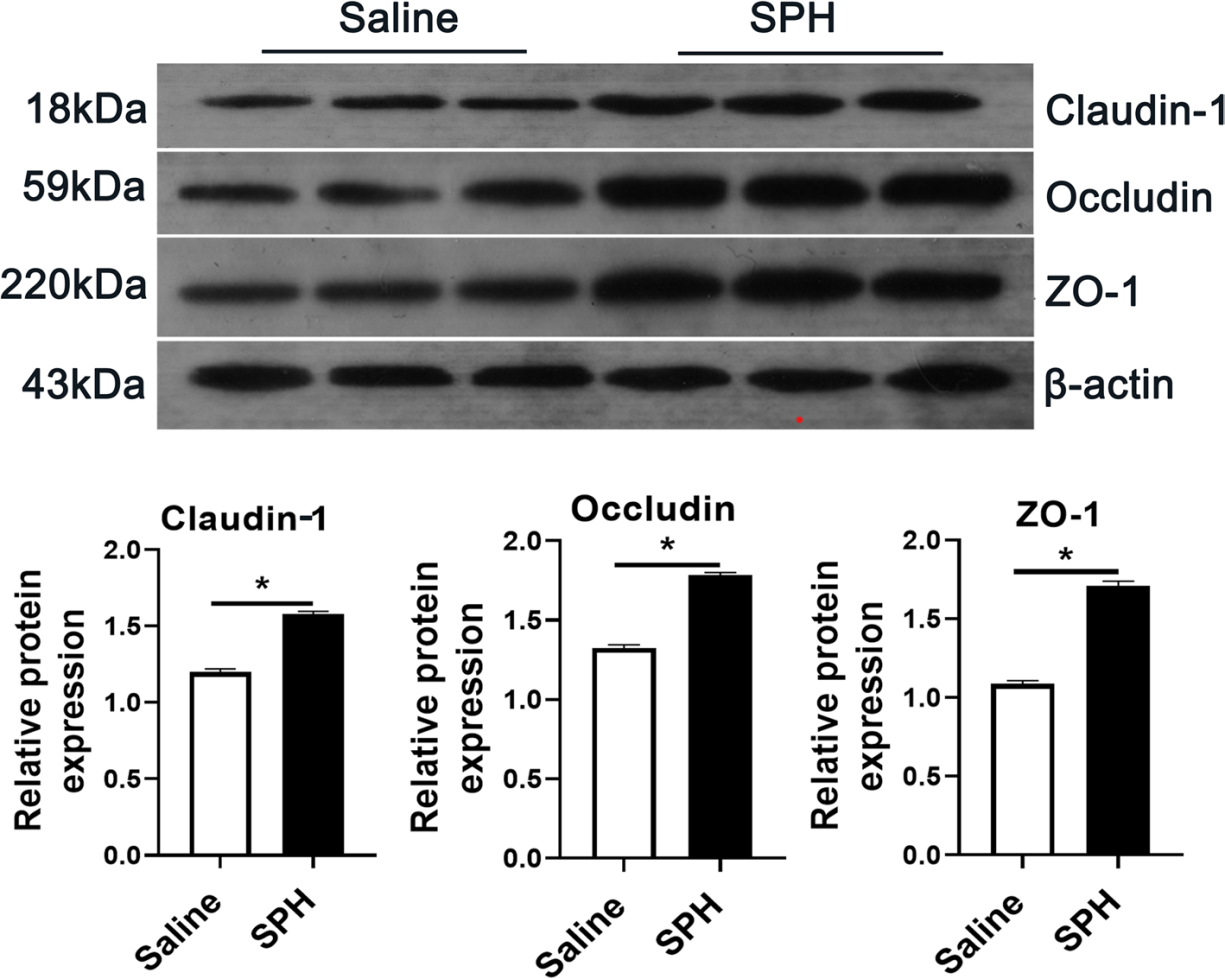
The expression of claudin-1, occludin, and ZO-1 in colonic mucosa. Values are mean ± SEM (*n* = 3). * *P* < 0.05

Compared with the saline group, the increased levels of TNF-α, IL-1β, IL-6, and IL-8 were found in the colonic mucosa of SPH pigs (*P* < 0.05, Fig. 3A). Correlation analysis showed that the concentration of pro-inflammatory cytokines was positively correlated with the abundance of eight different genera and the concentration of ammonia, cadaverine, putrescine, and isovalerate, and negatively correlated with the abundance of five different genera and the content of propionate and butyrate (Figs. 2D and 3B). Interestingly, SPH treatment also increased the protein expression of tight junction proteins—such as claudin-1, occludin, and ZO-1—in the colonic mucosa of treated pigs (*P* < 0.05, Fig. 4).

## Discussion

Previous studies revealed that high protein intake increases the body’s sense of fullness, thereby leading to a decrease in feed intake, which may reduce protein intake [11, 12]. When an animal model of HPD is established, it is necessary to consider whether there is an actual increase in protein intake of model animals. In this study, the duodenal infusion of SPH did not alter the average feed intake of pigs, indicating that the model actually increased the intestinal SPH level.

It revealed in this study that SPH infusion led to an increase in the species richness of colonic microbiota in growing pigs, including an increase in the abundance of protein-fermenting bacteria and a few harmful bacteria. For example, SPH infusion increased the abundance of certain genera, including *Erysipelotrichaceae*, *Prevotellaceae_uncultured*, *Prevotellaceae UCG-003*, and *Candidatus Saccharimonas*, which were involved in chronic inflammation [13–15], whereas a few species within the genera *Escherichia-Shigella*, *Dialister*, *Prevotella*, and *Veillonella* promoted by SPH treatment are regarded as opportunistic pathogens and are relevant to colitis [16–19]. On the other hand, *Escherichia-Shigella* and *Veillonella* are the main biogenic amine producers in the colon, while *Prevotella* and *Dialister* are associated with the production of branched chain fatty acids and ammonia, respectively [16–19]. The findings indicate that SPH increased the abundance of bacteria related to protein fermentation and chronic inflammation.

The abundance of propionate-producing bacteria *Anaerovibrio* [20] and butyrate-producing bacteria such as *Lachnospiraceae NK4A136 group* [21], *Coprococcus 3 *[22], *Lachnospiraceae_uncultured*, and *Lachnospiraceae UCG-002* [23] was markedly decreased in SPH treated pigs, while a reduction of propionate and butyrate concentrations in pig colon was revealed by SPH infusion. In fact, propionate and butyrate have shown anti-inflammatory effects and inhibitory effects of the expression of adhesion molecules and the production of chemokines [24]. The low concentrations of propionate and butyrate under SPH infusion may also suggest a decrease in immunity. Glutamate and lysine can be fermented by gut microbiota to produce butyrate [25]. The increased content of cadaverine from lysine decarboxylation due to SPH treatment observed in this study suggests an increased transformation of lysine towards cadaverine production rather than butyrate production. Moreover, a higher concentration of other amino acid metabolites, including putrescine, ammonia, and isovalerate was also shown after SPH infusion, which may imply that a potential cytotoxity, oxidative stress and inflammation were stimulated in the colon [26]. Marakova et al. reported that both putrescine and cadaverine levels can be used as potential biomarkers for inflammatory bowel disease (IBD) [27]. Collectively, our results demonstrated that SPH infusion increased the abundance of protein-fermenting bacteria and decreased the abundance of SCFAs-producing bacteria, leading to a higher level of biogenic amines and a lower level of SCFAs, which may bring a potential risk for colonic health.

The levels of TNF-α, IL-1β, IL-6, and IL-8 in the colonic mucosa of pigs were increased following SPH infusion. Lya et al. observed that an HPD caused diarrhoea in piglets by activating intestinal inflammation induced by NF-κB signalling [28]. Research in rats demonstrated that HPD increased the abundance of colonic pathogens and the concentration of protein fermentation products, leading to colon inflammation [5]. Therefore, the microbiota and metabolites under SPH intervention may contribute to colon inflammation in growing pigs, which is consistent with data from the correlation analysis of inflammatory cytokines with microbial communities and its metabolites.

A large body of literature reported that intestinal inflammation is largely related to the intestinal barrier function [29–31]. However, some of them revealed that in pigs only severe colon inflammation increased intestinal epithelial permeability through the disruption of the tight junction structure [31, 32]. Thus, the colon of pigs was proposed to have a mucosal adaption to maintain barrier function and epithelial homeostasis [3]. In our study, SPH infusion promoted the expression of tight junction proteins, including ZO-1, occludin, and claudin-1in the colonic mucosa, indicating that the increased SPH may contribute to maintaining colonic barrier integrity. Chen et al. evaluated the effects of different dietary protein levels (12%, 15%, and 18% protein) on the intestinal barrier function of growing pigs, and found that an appropriate increase in dietary protein concentration, such as 18%, can promote the expression of tight junction proteins in the colon [33]. Protein level was 21.4% in the SPH group in the study, higher than 18% in dietary protein, but we deduce it was not sufficient to induce severe inflammation so that the impaired barrier was not observed. Probably, the secretion of inflammatory cytokines stimulated by SPH intervention may cause only mild inflammation. However, mild colonic inflammation and related inflammatory cytokines can promote the repair and strengthening of the barrier [34]. Actually, researches in pigs associated with HPD, intestinal inflammation and barrier function are rare, while similar researches in rodents are numerous. Regarding to intestinal inflammation and barrier function, a range of protein contents are needed to be investigated in pigs for future study.

Moreover, the duodenal fistula model in this study is more suitable for our previous experiments in the small intestine. In the future, it is worth considering that a cecal fistula model is directly used to increase the colonic SPH level, and more indicators for the evaluation of colon inflammation and barrier function could be included.

## Conclusion

This study demonstrated that an increased SPH level in small intestine of pigs promoted colon inflammation, which was most likely attributed to the alteration in the composition and metabolism of colonic microbiota, such as a reduction of the abundance of propionate- and butyrate-producing bacteria, the content of propionate and butyrate, and an increase of the abundance of potentially pathogenic bacteria and protein-fermenting bacteria as well as the content of protein-fermented products such as putrescine, cadaverine, ammonia, and isovalerate, leading to the colonic inflammation without damaging the tight junction.

## Materials and methods

### SPH preparation

The SPH was prepared according to a previous method with slight modification [35]. In briefly, soy protein isolate (9010–10-0) (Yuanye Biotechnology, Shanghai, China) was suspended in 10% (w/v) distilled water, and porcine pepsin (Yuanye Biotechnology, S10027) hydrolysis was conducted at an enzyme-to-substrate ratio of 1:100 (w/w), pH 2.0, and 37 °C for 1 h. Thereafter, the pH of the solution was adjusted to 7.0 with 2 N NaOH to terminate the reaction. The obtained hydrolysate was stored at -20 °C and lyophilised in a FreeZone 4.5 L Freeze Dry System (Labconco Co., Kansas City, MO, USA) for further use.

### Animals and experimental procedures

Sixteen castrated pigs (Duroc × Landrace × Large White, aged 50 days) with an initial weight of 14.5 ± 0.2 kg were obtained from a commercial farm in Jiangsu Province, China. The pigs were kept in separate metabolic cages under a constant temperature of 25 ± 2 °C and given unlimited access to water and pig feed. After a week of acclimatisation, the pigs were fasted for 12 h before installing a simple T-cannula in the duodenum [36]. The length, width, and inner diameter of the T-cannula were 8.2, 10.0, and 1.5 cm, respectively. After the surgery, all pigs were hypodermically injected with ceftriaxone sodium and the wound and adjacent skin were disinfected with iodine tincture for one week (twice a day) to avoid potential infection. After they fully recovered from the duodenal fistula surgery over a two-week recuperation period, a two-week short-term experiment of SPH on the secretion of an intestinal satiety hormone was performed [10]. After a week of rest, all pigs were randomly allocated to the saline (control, *n* = 8) group and SPH group (*n* = 8) with no differences in body weight (35.2 ± 0.3 kg) and feed intake. The entire experiment was conducted over a period of 16 days, during which the pigs in the saline and SPH groups were infused with 10 ml sterile saline and 10 ml SPH solution (70 g/day), respectively, through a duodenal fistula at 8:00 am and 5:00 pm each day. The SPH solution was adjusted to pH 5.0, which is close to the native pH of porcine duodenum [37]. The basal diet in the experiment was designed based on the National Research Council (NRC) (2012) (Table 2). The feed consumption of each pig was recorded every day to calculate average feed intake. In addition, the body weights of all pigs were recorded on days 1 and 16 to determine average weight gain.

### Sample collection

After fasting for 12 h on day 16, all pigs were given general anaesthesia by an intravenous injection of sodium pentobarbital solution (40 mg/kg body weight), and were sacrificed by jugular exsanguination. The colons of all pigs were taken out within 5 min after slaughter and measured for length; fresh digesta from the colon was collected in a sterile tube and immediately stored at -20 °C for the analysis of microbiota and metabolites. The colon was weighed after removing all the digesta. In addition, a small piece of the colon tissue was collected and washed with phosphate buffered solution (PBS, pH 7.0); then, the colonic mucosa was scraped and immediately frozen in liquid nitrogen, and used to determine the concentration of inflammatory cytokines and the protein expression of tight junction proteins.

### Analysis of colonic microbial metabolites

The content of short-chain fatty acids (SCFAs) was determined using the method of Wang et al*.* [38]. Briefly, 1.5 ml of double distilled water was added into 0.4 g of digesta. The mixture was mixed and centrifuged (13,400 × g, 4℃, 10 min) in order to obtain supernatants. 1 ml supernatant and 200 μL of 25% (w/v) metaphosphoric acid were mixed and kept overnight at -20 °C. Then, the supernatant was centrifuged (13, 400 × g, 4℃, 10 min) and filtered with a 0.22 μL filter, and then measured with an Agilent 7890B gas chromatograph.

High-performance liquid chromatography (HPLC) was selected to detect the content of biogenic amines according to the method given by Yang et al. [39]. For this purpose, 0.6 g of digesta was weighed and 1.5 ml of trichloroacetic acid solution was added to precipitate the proteins and peptides. The sample was extracted with n-hexane and derived with dansyl chloride. Gradient elution of two solvents was carried out in the following manner: solvent A and solvent B were HPLC grade water and acetonitrile respectively, and the flow rate was set to 1.0 mL/min.

In addition, 0.1 g of digesta was prepared and acidified with 0.9 ml of 0.2 mol/L HCl for the determination of ammonia content using a spectrophotometer (UV-2450; Shimadzu, Tokyo, Japan) following the previous method [40].

### 16S rRNA sequencing

DNA from different colonic digesta was extracted using a DNA extraction kit (Qiagen, Hilden, Germany) following the manufacturer’s instructions. DNA concentration and purity were measured. The primers 515F and 907R were used to amplify the V4-V5 region of the 16S rRNA gene according to the previous methods [41]. Thereafter, the amplicon libraries were established using NEB Next®Ultra™DNA Library Prep Kit for Illumina (NEB, USA) based on the manufacturer’s illustrations. Then, the library was sequenced on an Illumina MiSeq platform. QIIME (version 1.70) was used to demultiplex and quality-filter the raw FASTQ files. Sequences with ≥ 97% similarity were classified as one operational taxonomic unit (OTU). The representative sequence of each OTU was selected, and the Ribosomal Database Project (RDP) classifier was used to annotate taxonomic information. The OTU table was refined to calculate alpha diversity. Beta diversity was calculated based on principal coordinates analysis (PCoA) and Unweighted Pair Group Method with Arithmetic Mean (UPGMA). Sequence alignment was performed with BLAST, and the feature sequences were annotated for each representative sequence using the SILVA database (Release 132) to determine the different taxonomies.

### Cytokine assay

The levels of interleukin (IL)-6, IL-1β, IL-8, IL-10, and tumour necrosis factor-α (TNF-α) in the colon mucosa supernatants were quantified using ELISA kits (Nanjing Jiancheng Bioengineering Institution, Nanjing, China) under manufacturer’s recommendations.

### Western blotting assay

The expression of tight junction proteins of the colon was determined by western blotting analysis based on the previous method [42]. The primary antibodies used include anti-zonula occludens 1 (ZO-1) (1:2,000, Abcam, ab211737), anti-occludin (1:1,000, Abcam, ab167161), anti-claudin-1 (1:1,000, CST, #5406), and anti-β-actin (1:1,000, Abcam, ab8226). Horseradish peroxidase-conjugated AffiniPure goat anti-mouse IgG (H + L) (1:5,000, Thermo Pierce, 31,160) or goat anti-rabbit IgG (H + L) (1:5,000, Jackson, Thermo Pierce, 31,210) were used as the secondary antibody. Image J software (NIH, Bethesda, MD, USA) was used to perform densitometry analysis.

### Statistical analysis

Data pertaining to the growth performance, intestinal index, microbial metabolites, protein expression of cytokines, and tight junction proteins were presented as mean ± standard error of mean (SEM). All data were compared in SPSS 20.0 (SPSS Inc., Chicago, USA) and graphs were drawn using GraphPad Prism 8.0.2 (La Jolla, CA, USA). Furthermore, the Student’s t-test was performed to analyze the difference significance, and significant differences were deemed when *P* < 0.05.

The alpha diversity was examined by the Student’s t-test. Different bacteria were compared by the Mann–Whitney U test with multiple comparisons adjusted by the Benjamini–Hochberg false discovery rate (FDR). The correlation among the abundance of different genera, the concentration of microbial metabolites, and mucosal inflammatory cytokines were analyzed according to Spearman’s correlation analysis (XLStat software; Addinsoft).

## Supplementary Information

**Additional file 1.**

## Data Availability

The 16S rRNA gene sequencing data on which the conclusions of the manuscript rely have been deposited in the National Center for Biotechnology Information (NCBI) database (accession number: SRP347652).

## Abbreviations

HPD: High-protein diets
SPH: Soy protein hydrolysate
SCFAs: Short-chain fatty acids
HPLC: High-performance liquid chromatography
OTUs: Operational taxonomic units
RDP: Ribosomal Database Project
PCoA: Principal coordinates analysis
UPGMA: Unweighted Pair Group Method with Arithmetic Mean
IL: Interleukin
TNF-α: Tumour necrosis factor-α
ZO-1: Zonula occludens 1
FDR: False discovery rate
ACE: Abundance-based coverage estimator
IBD: Inflammatory bowel disease

## References

[1] Gao J, Yin J, Xu K, Li T, Yin Y (2019). What is the impact of diet on nutritional diarrhea associated with gut microbiota in weaning piglets: a system review. Biomed Res Int.

[2] Wu Y, Jiang Z, Zheng C, Wang L, Zhu C, Yang X (2015). Effects of protein sources and levels in antibiotic-free diets on diarrhea, intestinal morphology, and expression of tight junctions in weaned piglets. Anim Nutr.

[3] Zhang H, Wielen NV, Hee BV, Wang J, Hendriks W, Gilbert M (2020). Impact of fermentable protein, by feeding high protein diets, on microbial composition, microbial catabolic activity, gut health and beyond in pigs. Microorganisms.

[4] Fan P, Li L, Rezaei A, Eslamfam S, Che D, Ma X (2015). Metabolites of dietary protein and peptides by intestinal microbes and their impacts on gut. Curr Protein Pept Sci.

[5] Mu C, Yang Y, Luo Z, Guan L, Zhu W (2016). The colonic microbiome and epithelial transcriptome are altered in rats fed a high-protein diet compared with a normal-protein diet. J Nutr.

[6] Russell WR, Gratz SW, Duncan SH, Holtrop G, Ince J, Scobbie L (2011). High-protein, reduced-carbohydrate weight-loss diets promote metabolite profiles likely to be detrimental to colonic health. Am J Clin Nutr.

[7] Richter JF, Pieper R, Zakrzewski SS, Gunzel D, Schulzke JD, Van Kessel AG (2014). Diets high in fermentable protein and fibre alter tight junction protein composition with minor effects on barrier function in piglet colon. Br J Nutr.

[8] Zhou L, Fang L, Sun Y, Su Y, Zhu W (2016). Effects of the dietary protein level on the microbial composition and metabolomic profile in the hindgut of the pig. Anaerobe.

[9] Cone JW, Jongbloed AW, Van Gelder AH, de Lange L (2005). Estimation of protein fermentation in the large intestine of pigs using a gas production technique. Anim Feed Sci Tech.

[10] Wang L, Ding L, Zhu W, Hang S (2021). Soybean protein hydrolysate stimulated cholecystokinin secretion and inhibited feed intake through calcium-sensing receptors and intracellular calcium signalling in pigs. Food Funct.

[11] Kohanmoo A, Faghih S, Akhlaghi M (2020). Effect of short- and long-term protein consumption on appetite and appetite-regulating gastrointestinal hormones, a systematic review and meta-analysis of randomized controlled trials. Physiol Behav.

[12] Moon J, Koh G (2020). Clinical evidence and mechanisms of high-protein diet-induced weight loss. J Obes Metab Syndr.

[13] Dinh DM, Volpe GE, Duffalo C, Bhalchandra S, Tai AK, Kane AV (2015). Intestinal microbiota, microbial translocation, and systemic inflammation in chronic HIV infection. J Infect Dis.

[14] Sun L, Jia H, Li J, Yu M, Yang Y, Tian D (2019). Cecal gut microbiota and metabolites might contribute to the severity of acute myocardial ischemia by impacting the intestinal permeability, oxidative stress, and energy metabolism. Front Microbiol.

[15] Camelo-Castillo AJ, Mira A, Pico A, Nibali L, Henderson B, Donos N (2015). Subgingival microbiota in health compared to periodontitis and the influence of smoking. Front Microbiol.

[16] Yang Y, Wu H, Dong S, Jin W, Han K, Ren Y (2018). Glycation of fish protein impacts its fermentation metabolites and gut microbiota during in vitro human colonic fermentation. Food Res Int.

[17] Larsen JM (2017). The immune response to Prevotella bacteria in chronic inflammatory disease. Immunology.

[18] De Cruz P, Kang S, Wagner J, Buckley M, Sim WH, Prideaux L, et al. Association between specific mucosa-associated microbiota in Crohn’s disease at the time of resection and subsequent disease recurrence: a pilot study. J Gastroen Hepatol. 2015;30(2):268–78. 10.1111/jgh.12694 25087692

[19] Chen L, Li R, Wang Z, Zhang Z, Wang J, Qiao Y (2022). Lactate-utilizing bacteria ameliorates DSS-induced colitis in mice. Life Sci.

[20] Xia X, Li G, Ding Y, Ren T, Zheng J, Kan J (2017). Effect of whole grain qingke (tibetan hordeum vulgare l. zangqing 320) on the serum lipid levels and intestinal microbiota of rats under high-fat diet. J Agric Food Chem.

[21] Ma L, Ni Y, Wang Z, Tu W, Ni L, Zhuge F (2020). Spermidine improves gut barrier integrity and gut microbiota function in diet-induced obese mice. Gut Microbes.

[22] Ma T, Villot C, Renaud D, Skidmore A, Chevaux E, Steele M (2020). Linking perturbations to temporal changes in diversity, stability, and compositions of neonatal calf gut microbiota: prediction of diarrhea. Isme J.

[23] Mancabelli L, Milani C, Lugli GA, Turroni F, Mangifesta M, Viappiani A (2017). Unveiling the gut microbiota composition and functionality associated with constipation through metagenomic analyses. Sci Rep.

[24] Kleuskens M, Haasnoot ML, Herpers BM, Ampting M, Bredenoord AJ, Garssen J (2021). Butyrate and propionate restore interleukin 13-compromised esophageal epithelial barrier function. Allergy.

[25] Martin-Gallausiaux C, Marinelli L, Blottiere HM, Larraufie P, Lapaque N (2021). SCFA: mechanisms and functional importance in the gut. Proc Nutr Soc.

[26] Xia J, Fan H, Yang J, Song T, Pang L, Deng H (2021). Research progress on diarrhoea and its mechanism in weaned piglets fed a high-protein diet. J Anim Physiol Anim Nutr (Berl).

[27] Marakova K, Piestansky J, Zelinkova Z, Mikus P (2020). Simultaneous determination of twelve biogenic amines in human urine as potential biomarkers of inflammatory bowel diseases by capillary electrophoresis - tandem mass spectrometry. J Pharm Biomed Anal.

[28] Lya B, Jla C, Mw A, Qw A, Jl A, Nd C (2021). Dietary high protein-induced diarrhea and intestinal inflammation by activation of NF-κB signaling in piglets. Animal Nutrition.

[29] Gao Y, Davis B, Zhu W, Zheng N, Meng D, Walker WA (2021). Short-chain fatty acid butyrate, a breast milk metabolite, enhances immature intestinal barrier function genes in response to inflammation in vitro and in vivo. Am J Physiol Gastrointest Liver Physiol.

[30] Gasaly N, de Vos P, Hermoso MA (2021). Impact of bacterial metabolites on gut barrier function and host immunity: a focus on bacterial metabolism and its relevance for intestinal inflammation. Front Immunol.

[31] Zeng Y, Wang Z, Zou T, Chen J, Li G, Zheng L (2021). Bacteriophage as an alternative to antibiotics promotes growth performance by regulating intestinal inflammation, intestinal barrier function and gut microbiota in weaned piglets. Front Vet Sci.

[32] Grosheva I, Zheng D, Levy M, Polansky O, Lichtenstein A, Golani O (2020). High-throughput screen identifies host and microbiota regulators of intestinal barrier function. Gastroenterology.

[33] Chen X, Song P, Fan P, He T, Jacobs D, Levesque CL (2018). Moderate dietary protein restriction optimized gut microbiota and mucosal barrier in growing pig model. Front Cell Infect Microbiol.

[34] Luissint AC, Parkos CA, Nusrat A (2016). Inflammation and the intestinal barrier: leukocyte-epithelial cell interactions, cell junction remodeling, and mucosal repair. Gastroenterology.

[35] Kim SS, Ahn C, Moon SW, Je J (2018). Purification and antioxidant activities of peptides from sea squirt (Halocynthia roretzi) protein hydrolysates using pepsin hydrolysis. Food Biosci.

[36] Jongbloed AW, Mroz Z, Kemme PA (1992). The effect of supplementary Aspergillus niger phytase in diets for pigs on concentration and apparent digestibility of dry matter, total phosphorus, and phytic acid in different sections of the alimentary tract. J Anim Sci.

[37] Abello J, Corring T, Laplace JP (1987). Contribution of bile and pancreatic juice to the control of pH in the pig duodenum. Reprod Nutr Dev.

[38] Wang XF, Mao SY, Liu JH, Zhang LL, Cheng YF, Jin W (2011). Effect of the gynosaponin on methane production and microbe numbers in a fungus-methanogen co-culture. J Anim Feed Sci.

[39] Zhang Y, Yu K, Chen H, Su Y, Zhu W (2018). Caecal infusion of the short-chain fatty acid propionate affects the microbiota and expression of inflammatory cytokines in the colon in a fistula pig model. Microb Biotechnol.

[40] Nyachoti CM, Omogbenigun FO, Rademacher M, Blank G (2006). Performance responses and indicators of gastrointestinal health in early-weaned pigs fed low-protein amino acid-supplemented diets. J Anim Sci.

[41] Sun Y, Zhou L, Fang L, Su Y, Zhu W (2015). Responses in colonic microbial community and gene expression of pigs to a long-term high resistant starch diet. Front Microbiol.

[42] Meng Y, Zhang J, Zhang F, Ai W, Zhu X, Shu G (2017). Lauric acid stimulates mammary gland development of pubertal mice through activation of GPR84 and PI3K/Akt signaling pathway. J Agric Food Chem.

