# Supplementary material for: Effects of the increased protein level in small intestine on the colonic microbiota, inflammation and barrier function in growing pigs
Source: BMC Microbiol. 2022 Jul 6;22:172. doi: 10.1186/s12866-022-02498-x (PMC9258065; doi:10.1186/s12866-022-02498-x)
Supplement: Supplementary file 1 — Additional file 1. [file 12866_2022_2498_MOESM1_ESM.docx]

**Title:** **Effects of the increased protein level in small intestine on the colonic microbiota, inflammation and barrier function in growing pigs**

Zhongxin Li^1^, Liren Ding^2^, Weiyun Zhu^1^, Suqin Hang*^1^


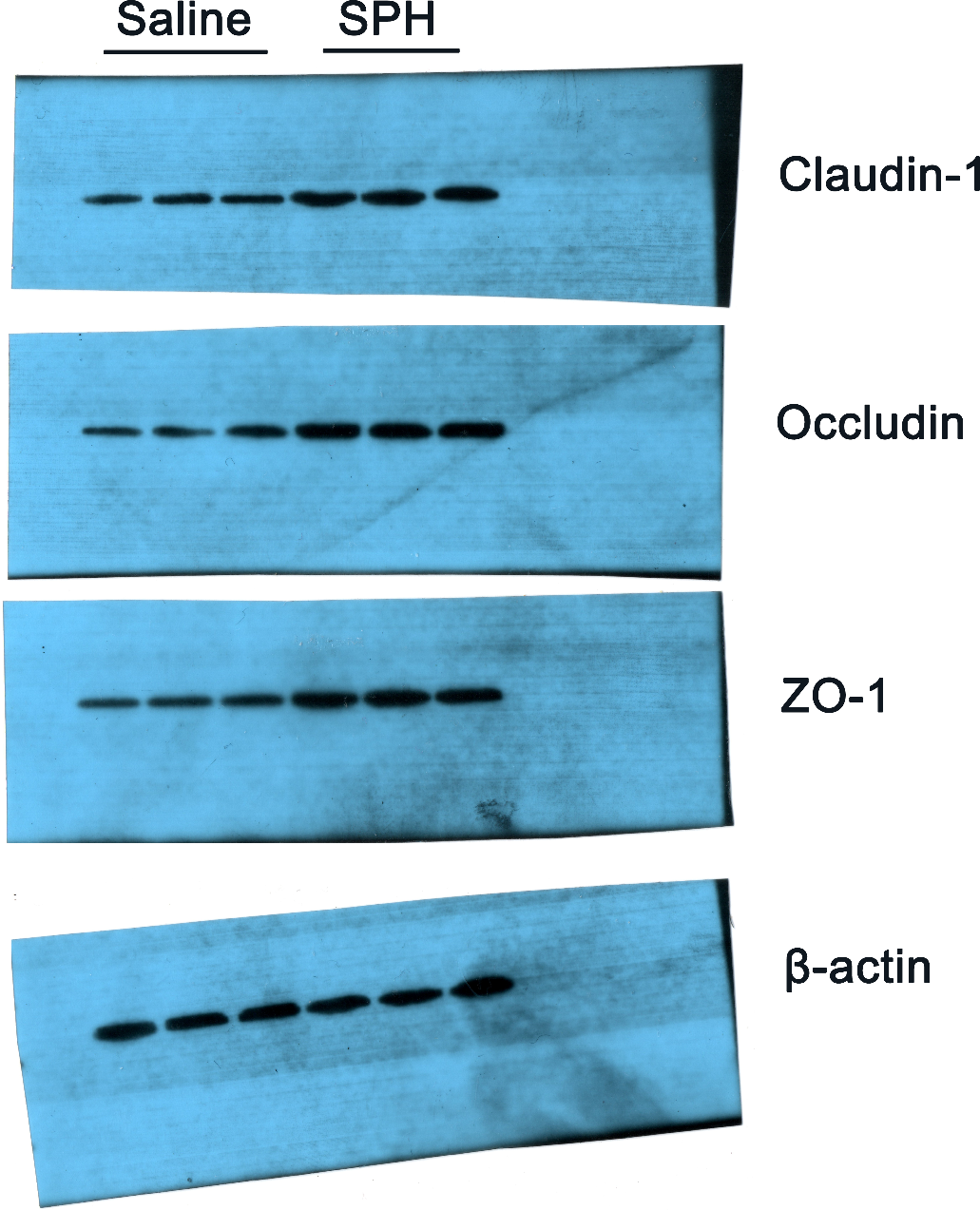


**Figure. S1** Original images of Figure.4. The samples derived from the same experiment and gels/blots were processed in parallel.
